# Sustainable Xanthine-Grafted Alginate Biosensing Platform for Metabolic Disorder Diagnostics

**DOI:** 10.1021/acsomega.6c02447

**Published:** 2026-06-01

**Authors:** Angelo Ferlazzo, Erika Saccullo, Giulia Sambataro, Elena Bruno, Manuel David Montaño, Vincenzo Abbate, Salvatore Failla, Venerando Pistarà, Antonino Gulino, Antonio Rescifina, Vincenzo Patamia, Giuseppe Floresta

**Affiliations:** a Department of Chemical Sciences, 9298 University of Catania, Viale Andrea Doria 6, Catania 95125, Italy; b Department of Drug and Health Sciences, 9298 University of Catania, Viale Andrea Doria 6, Catania 95125, Italy; c Department of Biomedical and Biotechnological Sciences (Biometec), University of Catania, Via Santa Sofia 97, Catania 95123, Italy; d Department of Physics and Astronomy “Ettore Majorana”, University of Catania, via S. Sofia 64, Catania 95123, Italy; e London Metallomics Facility, King’s College London, London SE19NH, U.K.; f Department of Analytical, Environmental & Forensic Sciences, Faculty of Life Sciences & Medicine, King’s College London, London SE19NH, U.K.

## Abstract

This work describes the development of an efficient, durable, and highly sustainable electrochemical biosensing platform for the detection of uric acid (UA), sodium alginate (Alg), which is functionalized with an allyl derivative of xanthine to yield AlgX. The entire synthesis process is notably eco-biocompatible, utilizing water as the solvent and green reagents, such as H_2_O_2_ and ascorbic acid. AlgX was subsequently complexed with Cu^2+^ ions to form AlgXCu, resulting in an active material with a very low metal content, calculated to be only 4.8% through inductively coupled plasma mass spectrometry. Electrochemical characterization of the AlgXCu/SPCE (screen-printed carbon electrode) sensor demonstrated good performance for UA detection. Differential pulse voltammetry results show a high sensitivity of 14.28 μA μM^–1^ cm^–2^ and a remarkably low limit of detection (LOD) of 0.025 μM. This LOD is a good value for copper-based sensors, even compared to those with a higher copper content. Furthermore, the sensor exhibited very good selectivity and the capability to detect UA and dopamine (DA) simultaneously. Its applicability was validated in synthetic saliva and urine samples, demonstrating high accuracy and recovery rates (recovery between 97.9 and 105%) and excellent reproducibility (RSD ≤ 2.4%).

## Graphical abstract

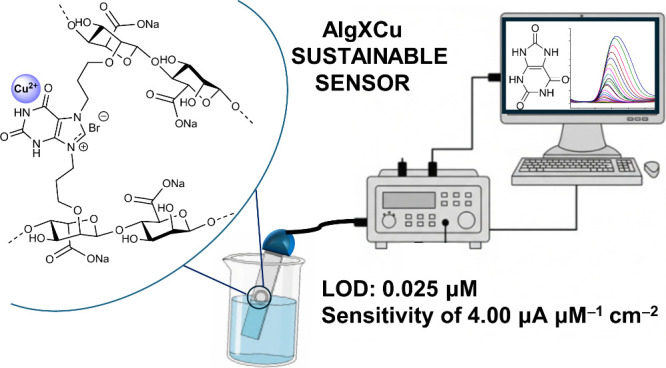

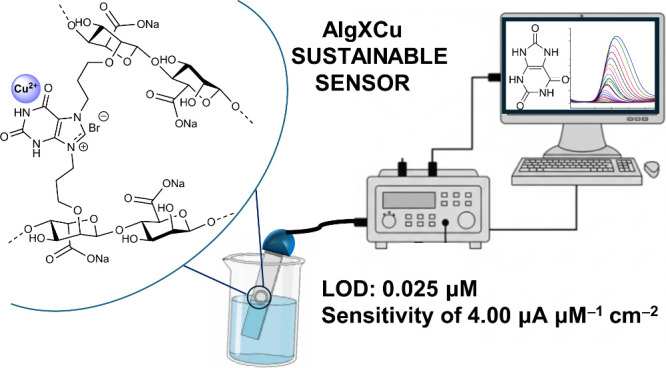

## 1 Introduction

Biosensors, particularly those fabricated from naturally derived materials and employing sustainable processes, represent a promising frontier in modern medical diagnostics. Their ability to detect specific biomarkers with high sensitivity and selectivity is crucial for early diagnosis and effective management of numerous pathologies. Among these, Lesch-Nyhan Syndrome (LND), a rare and severe X-linked genetic disorder, presents a compelling case in which precise biomarker identification is vital.

LND is characterized by a deficiency of the hypoxanthine-guanine phosphoribosyltransferase (HPRT) enzyme, resulting in a pathological accumulation of uric acid.^1^ The resulting hyperuricemia manifests as urate nephropathy, gout, and urolithiasis. Beyond these metabolic complications, LND is uniquely associated with severe neurological symptoms, including dystonia, spasticity, and, most devastatingly, compulsive self-injurious behaviors.^2^ These neurological symptoms have been correlated with significant alterations in neurotransmitter metabolism, notably a marked reduction in dopamine levels and its primary metabolite, homovanillic acid (HVA), in the central nervous system.^3^

The simultaneous presence of elevated uric acid levels and a dopamine deficiency (evidenced by reduced HVA in cerebrospinal fluid) makes these two compounds critical biomarkers for the diagnosis and monitoring of LND. In healthy individuals, typical plasma uric acid levels range from 2.5 to 7.0 mg/dL (149–416 μmol/L), while in LND patients, levels are significantly elevated, often exceeding 10 mg/dL (595 μmol/L).^4,5^ Similarly, while cerebrospinal fluid (CSF) HVA levels in healthy subjects can typically range from 100 to 300 ng/mL (550–1650 pmol/mL) depending on age, LND patients exhibit a profound reduction, with HVA levels often 60–90% lower than those of healthy controls, reflecting severe cerebral dopamine dysfunction.^3,6^ Timely and accurate detection of these alterations is fundamental for early intervention and the development of targeted therapies. However, current diagnostic methodologies, often based on complex and invasive laboratory techniques (such as lumbar puncture for CSF analysis), underscore the need for alternative, faster, less invasive, and more accessible diagnostic approaches.

In this context, the development of selective biosensors for uric acid and dopamine emerges as a highly valuable solution. Such devices could offer in situ, real-time detection with minimal sample volumes, revolutionizing diagnostics and patient monitoring for LND. The importance of these biosensors is further amplified by the possibility of fabricating them from naturally derived materials through sustainable synthesis processes. Polysaccharides, for example, provide an excellent platform for this application due to their biocompatibility, biodegradability, abundance, and tunable features.^7–9^

Alginic acid, a linear polysaccharide extracted from brown algae, has been widely explored for biosensor fabrication due to its excellent film-forming and hydrogel-forming properties, as well as its ability to immobilize enzymes and nanomaterials.^10^ Similarly, chitosan, a deacetylated derivative of chitin found in crustacean shells, is highly regarded for its positive charge, mucoadhesive properties, and good film-forming ability, making it ideal for creating sensitive and stable sensing interfaces.^11^ Biosensors based on alginic acid or chitosan can be designed to incorporate specific recognition elements (e.g., enzymes like uricase for uric acid detection, or aptamers/molecularly imprinted polymers for dopamine) within their matrix, providing enhanced selectivity and sensitivity.^12,13^

The utilization of biopolymers and other biocompatible natural materials not only reduces the environmental footprint of production but also enhances the biocompatibility and biodegradability of the devices, crucial aspects for medical applications.^14,15^ Research in this direction not only promotes advanced diagnostics for rare diseases like LND but also aligns with the principles of green chemistry and sustainable development, outlining a future where medical technology serves both human health and the planet. Despite the potential of biopolymers, the development of eco-friendly sensors that combine high sensitivity with low metal loading remains a challenge. This work aims to develop a sustainable electrochemical platform based on a novel xanthine-functionalized alginate matrix (AlgX) complexed with Cu^2+^. The novelty lies in the synergistic coordination that allows for a record-low copper content (0.5%) while achieving an exceptional LOD of 0.025 μM for Uric Acid. The AlgX system plays a key role both in chelating copper, which would otherwise not be effectively retained by nonfunctionalized Alg, and in promoting adhesion to the electrode surface, since AlgX is less soluble in aqueous solution than Alg alone. By utilizing an entirely green synthetic route in water, this study provides a highly efficient and noninvasive tool for the simultaneous detection of UA and DA, specifically tailored for the clinical needs of Lesch-Nyhan Syndrome monitoring.

## 2 Materials

### 2.1 General Information

All chemicals were purchased from Merck, Fisher Scientific, and VWR. ^1^H NMR spectra were recorded at 300 K on a Varian UNITY Inova spectrometer (500 MHz), using D_2_O as the solvent. Chemical shift (δ) values were given in ppm.

### 2.2 Synthesis of Materials

#### 2.2.1 Synthesis of AlgX

The procedure was carried out by modifying a method described in the literature.^16^ Sodium alginate (Alg) (100 mg) was dissolved in 6.2 mL of a 2% acetic acid solution in a double-neck flask under a nitrogen flow. Once solubilized, 100 mg of allyl xanthine (X), 12.5 mg of ascorbic acid, and 200 μL of 0.25 M H_2_O_2_ were added, and the mixture was stirred at room temperature for 5 h. After 5 h, the crude reaction mixture was dialyzed (1000 Da) overnight. The resulting solution was evaporated under vacuum to obtain 150 mg of a transparent film (see Figure S1).

#### 2.2.2 Synthesis of AlgXCu

To a dispersion of AlgX (40 mg) in H_2_O (2 mL), CuCl_2_ (50 mg) was added.^17,18^ The resulting solution was stirred at 55 °C overnight. The crude product was centrifuged and washed several times with deionized H_2_O to remove excess uncomplexed metal ions, yielding 30 mg of a greenish solid.

### 2.3 Characterization

#### 2.3.1 Infrared Spectroscopy

FTIR-ATR analyses were conducted using an FTIR Agilent Cary 630 equipped with an ATR sampling module. Thin films of the samples were applied to the ATR crystal and pressed gently. The results were derived from 512 scans acquired in the 4000–500 cm^–1^ range with a resolution of 2 cm^–1^ at room temperature.

#### 2.3.2 Thermogravimetric Analysis

Thermal gravimetric analysis (TGA) was performed under 1 atm of prepurified nitrogen at a heating rate of 10 °C/min over the temperature range 50–900 °C. The instrument used is the PerkinElmer TGA 4000.

#### 2.3.3 Scanning Electron Microscopy-Energy-Dispersive X-ray Spectroscopy

The synthesized material’s morphology was analyzed by scanning electron microscopy using a Dual Beam Focused ion beam Versa 3D LoVac DualBeam in secondary electron mode using a 5 keV electron beam. The sample was also in situ analyzed by energy-dispersive X-ray spectroscopy (EDS) using a 20 keV electron beam. Before analysis, samples were dispersed on a conductive carbon tape and sputtered with 5 nm of Au to ensure proper conductivity during measurements.

#### 2.3.4 Evaluation of Mean Particle Size and Polydispersity Index

To evaluate the mean particle size (Z-ave) and polydispersity index (PdI) of AlgX and AlgXCu, they were solubilized/suspended in water (1 mg/mL) and analyzed using Photon Correlation Spectroscopy (PCS) with a Zetasizer Nano S90 instrument (Malvern Instruments, Malvern, UK). The instrument was set to a detection angle of 90° and a 4 mW He–Ne laser operating at 633 nm, at 25 °C. Three sets of measurements were used in the sample analysis, and the mean size ± standard deviation (SD) was reported as the result.

#### 2.3.5 Inductively Coupled Plasma Mass Spectrometry

To quantify the concentration of copper in the sample, a known amount of the material was weighed into a 15 mL polypropylene centrifuge tube. Two mL of concentrated nitric acid (trace metal grade, Fisher Scientific) was added, and the sample was digested in an aluminum hot block for 1 h at 60 °C until fully dissolved. The digest was then diluted into 2% nitric acid for analysis by ICP-MS.

The ICP-MS used was a NexION 5000 (PerkinElmer) equipped with a type-C MiraMist nebulizer and a baffled cyclonic spray chamber. Eight external calibrants ranging from 0 to 500 μg/L were prepared in 2% nitric acid. An internal standard comprised of 50 μg/L Ga was used to correct for instrumental drift. Mass ^63^Cu was monitored in dynamic reaction cell (DRC) mode using ammonia as the reaction gas to improve detection limits. Additional operating parameters for the instrument can be found in Table S1.

#### 2.3.6 Electrochemical Measurements

Cyclovoltammetry (CV), electrical impedance spectroscopy (EIS), differential pulse voltammetry (DPV), and chronoamperometric (CA) measurements were performed using a DropSens μStat-i 400s potentiostat/galvanostat, equipped with Dropview 8400 software. A 0.1 M phosphate-buffered saline (PBS) solution, pH 7.4, was used for electrochemical measurements. CV was performed at a scan rate of 50 mV/s over the potential range of −0.4 to 1.1 V, using PBS, 10 mM potassium ferricyanide (K_3_[Fe­(CN)_6_]), and 0.1 M KCl standard solutions. EIS tests were conducted using 10 mM potassium ferricyanide (K_3_[Fe­(CN)_6_]) and 0.1 M KCl standard solutions in the 0.1–10^5^ Hz frequency range, amplitude 10 mV, and applied potential 0.25 V.^19^

DPV tests were conducted using optimized potential step (E_step_) of 0.03 V, potential pulse (E_puls_) of 0.09 V, and time pulse (T_pul_) of 200 ms, with a scan rate of 40 mV/s in the −0.4 to 0.7 V potential range; CA curves were obtained by recording the oxidation current at a constant potential of 0.35 V, and CV analysis, under the same conditions as above-described with −0.2 to 1.0 V potential range. At the same time, an appropriate volume (0.1–800 μL) of 10 mM uric acid (UA) and dopamine (DA) solution was added to the electrolyte solution (PBS 0.1 M) under magnetic stirring.

Measurements were made using a commercial reference Screen-Printed Carbon Electrode (SPCE) from Metrohm DropSens and a working SPCE modified with AlgXCu by depositing 20 μL of its suspension (5 mg in 1 mL of distilled water).

The resulting sensors (AlgXCu/SPCE) were dried at room temperature for 24 h under an inert atmosphere (Scheme 1).^20^

**Scheme 1:**
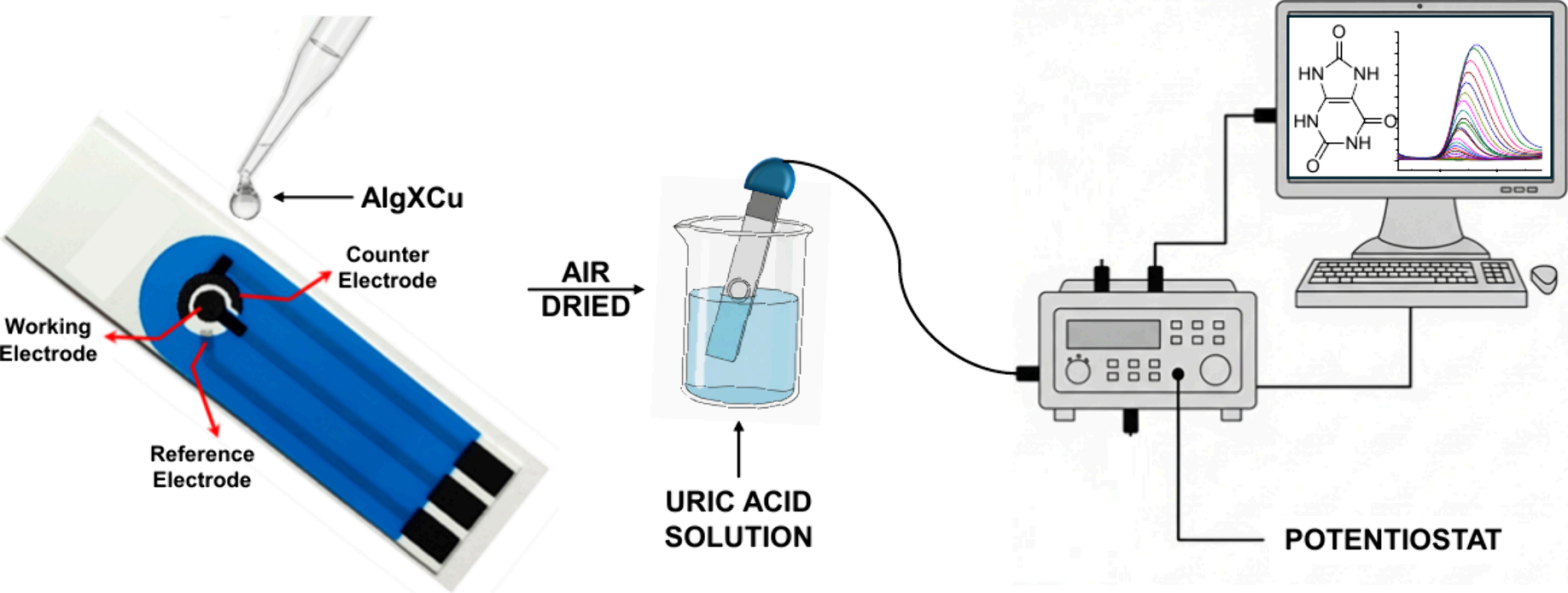
Schematic Setup of Electrochemical Experiments

The sensor sensitivity (S) was always calculated (eq 1) as the ratio between the slope (m) of the calibration line and the geometric surface area (A) of the non modified SPCE electrode (0.125 cm^2^).^21^ The LOD was calculated by multiplying the ratio between the standard error value of the intercept (SE_int_) and the slope (m) of the calibration line by 3.3 (eq 2).^22^

$$S=\frac{m}{A}$$ 1

$$\mathrm{LOD}=3.3\times \frac{SE_{\mathrm{int}}}{m}$$ 2

#### 2.3.7 Electrochemical Measurements for Synthetic Saliva and Urine

To investigate the ability of the AlgXCu/SPCE sensor (vide infra) to detect UA and DA in synthetic matrices, we performed DPV analysis before and after spiking with 100 and 1000 μM UA and DA, using the addition method.^23,24^

We investigated UA and DA concentrations in synthetic saliva and synthetic urine using DPV with the already-optimized potential step, potential pulse, time pulse, and scan rate. Synthetic urine and saliva were prepared following a methodology reported in the literature.^25,26^ In synthetic saliva, a known amount of UA (40 μM) was added, a typical value under normal physiological conditions (∼1.2 × 10^–5^–∼ 5.9 × 10^–5^ mol/L).^27^

Furthermore, synthetic urine samples were used without sample pretreatment, while synthetic saliva samples were diluted 40 times with 0.1 M PBS before measurement, according to a protocol already reported for this type of investigation.^28^

## 3 Results and Discussion

### 3.1 Synthesis

This work aims to develop an efficient and durable electrochemical sensor for uric acid detection using a sustainable, eco-biocompatible platform based on natural, readily prepared materials. The material consists of a polysaccharide matrix, such as sodium alginate, functionalized with an allyl derivative of xanthine via a sustainable synthetic process using water as the solvent, H_2_O_2_, and ascorbic acid (Figure 1). This procedure produces a transparent film that is then resolved to carry out the Cu^2+^ complexation reaction, exploiting xanthine for chelation, which is helpful for the electrochemical detection of uric acid.

**Figure 1:**
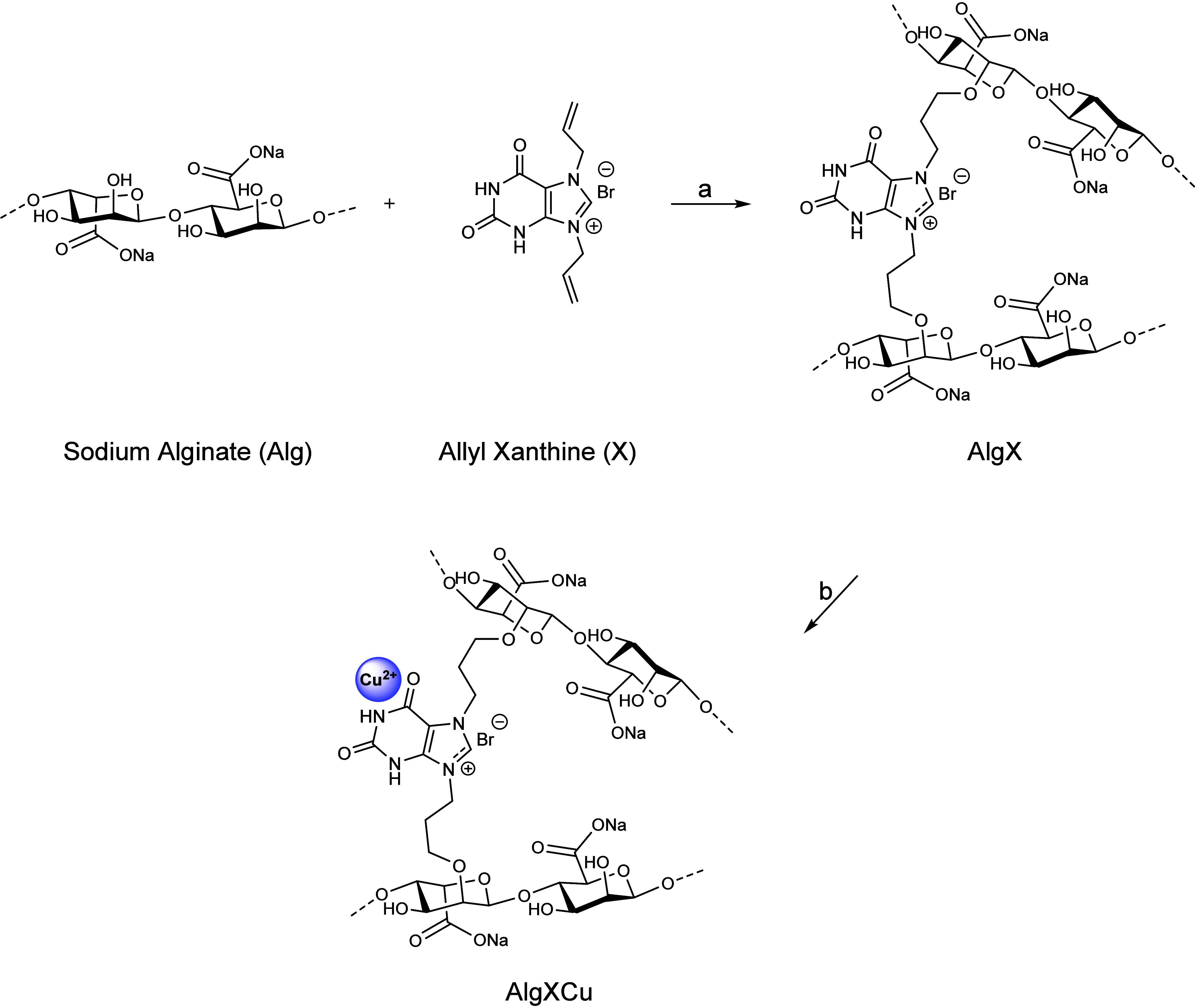
Reaction scheme for synthesizing AlgXCu material: (a) CH_3_COOH 2%, ascorbic acid, H_2_O_2_, r.t., 5 h; (b) CuCl_2_, 55 °C, overnight.

### 3.2 Analytical Characterization

Using ^1^H NMR analysis (Figure 2), the starting material, Alg (blue line), was compared with AlgX (black line), synthesized using the new procedure (black line), to verify grafting and the proper formation of the covalent bond between the polysaccharide backbone and xanthine. The ^1^H NMR spectrum of Alg (Figure 2, blue line) shows typical polysaccharide signals attributed to aliphatic hydrogens within the sugar monomers, ranging from 3.5 to 5.2 ppm.^29^ The AlgX spectrum (Figure 2, black line) clearly reveals the presence of xanthine through new peaks typical of the xanthine imidazole group at 7.98 and 7.94 ppm, as well as additional peaks at 2.71, 2.47, and 2.06 ppm. At 6.10 ppm, a signal attributable to the allylic double bonds is observed.^30,31^ These last signals represent the methylene protons and indicate the formation of new carbon–oxygen bonds.^31,32^

**Figure 2:**
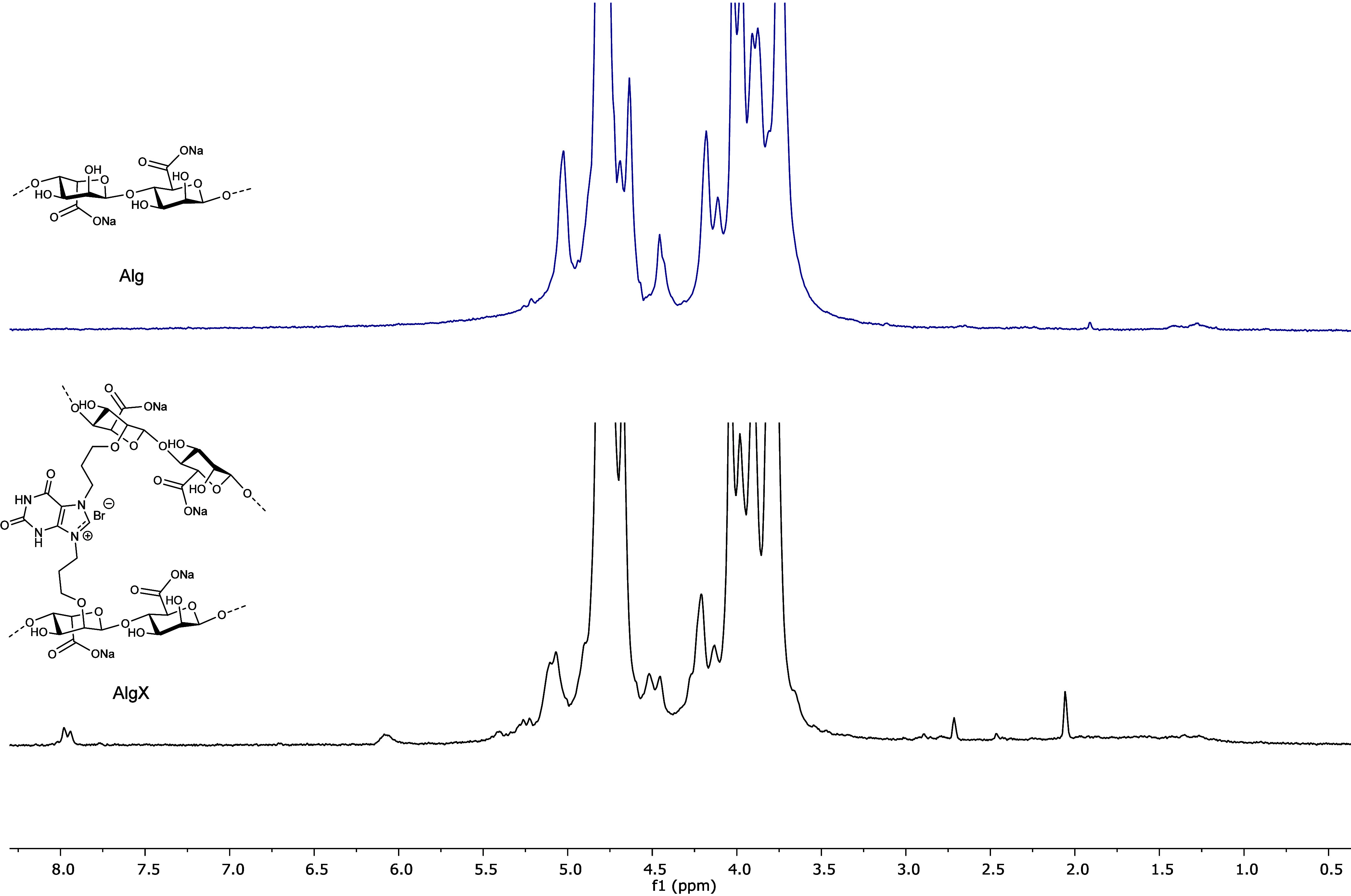
Stacked ^1^H NMR spectra in D_2_O of sodium alginates (Alg) (blue line) and AlgX (black line).

Further confirmation of the successful functionalization of Alg with X is evident in the FTIR spectrum shown in Figure 3. Together with the characteristics of the polysaccharide chain of alginic acid 1) at 1025 cm^–1^ attributed to symmetric C–O–C stretching, 2) at 1593 cm^–1^ to the C–O carboxylate stretching vibration, 3) at 2924, to the aliphatic C–H bond, and 4) at 3250 cm^–1^ to the stretching of the OH groups (Figure 2, black line),^29,33,34^ distinct signals relating to xanthine are also evident 1) at 1330 cm^–1^ to the stretching of the C–N bond, 2) at 1700 and 1600 cm^–1^ to the stretching of the C=O and the C=C, respectively; 3) at 3000 and 2883 cm^–1^ for the stretching of the single aliphatic C–H bond of the alkyl chain linked to xanthine; 4) at 3129 cm^–1^ for the stretching of the single aromatic C–H bond and finally the new stretching signal at 1200 cm^–1^ verifies the formation of the C–O bond established during the functionalization reaction (Figure 3, red line).^31,35^ The IR spectrum also clearly shows the formation of the complex with copper, as evidenced by changes in the carbonyl group signals and the site of interaction with copper (Figure 3, line blue).^36,37^

**Figure 3:**
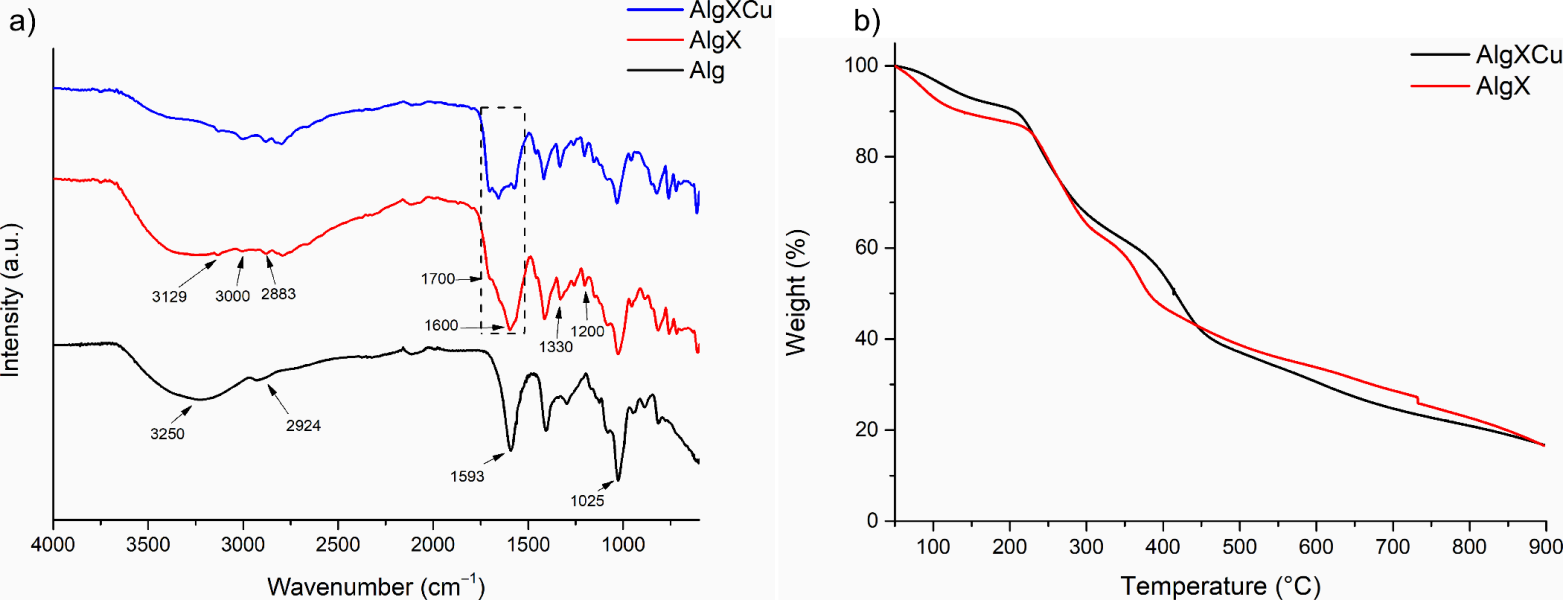
(a) Stacked FTIR spectra Alg (black line), AlgX (red line), and AlgXCu (blue line); (b) Thermogravimetric curves of AlgXCu (black line) and AlgX (red line).

The thermal stability and degradation patterns of AlgX and AlgXCu were evaluated via thermogravimetric analysis (Figure 3b). Although both materials exhibit a multistage weight loss, the incorporation of copper ions significantly alters the thermal profile, as detailed in Table 1. Specifically, AlgXCu shows a more pronounced mass loss in the 150–550 °C range compared to AlgX. This behavior is attributed to the catalytic effect of Cu^2+^ ions, which facilitates the early cleavage of glycosidic bonds and the formation of intermediate metal oxalate fragments.^38^ These intermediates subsequently decompose into stable metal oxides at higher temperatures, resulting in the final residues observed at 900 °C.

**Table 1:** Mass Loss Percentages of AlgX and AlgXCu

**Mass loss%**					
**SAMPLE**	*T* < 150 **°C**	**150 °C <** *T* < 350 **°C**	**350 °C <** *T* < 550 **°C**	**550 °C <** *T* < 900 **°C**	Residue %
AlgXCu	7.60	31.26	27.47	16.90	16.77
AlgX	10.74	30.92	20.91	21.33	16.10

The sizes of the AlgX and AlgXCu aggregates in aqueous suspension were analyzed using PCS (Table 2). The results show that AlgX has an average size in the nanometer range (403.9 nm). In comparison, the complexation of Cu in AlgXCu led to a slight increase in size to 496 nm,^39^ accompanied by a decrease in polydispersity (from 0.529 to 0.332).

**Table 2:** Mean size (Z-ave) and Polydispersity Index (PdI) of AlgX and AlgXCu

**Sample**	Z-ave (nm) ± SD	PDI ± SD
AlgX	403.9 ± 7.3	0.529 ± 0.05
AlgXCu	496.2 ± 24.6	0.332 ± 0.07

To quantify the amount of complexed Cu^2+^, the sample was digested and then analyzed by ICP-MS. After accounting for dilution factors and the sample mass, the concentration of copper was determined to be 48.906 g Cu/kg sample (4.8%).

Figure 4 shows SEM images of AlgXCu at different magnifications. From the various magnifications, it is evident that the material has a compact morphology, which is highly functional for the sensor platform to remain attached to the electrode for as many cycles as possible. When combined with EDS analysis, it also allows us to confirm the massive presence of C, N, and O, as evident from the maps in Figure 5a–d. Furthermore, the spectrum shown in Figure 5e indicates the occurrence of copper in the material, thereby supporting the findings of inductively coupled plasma mass spectrometry. At the same time, residual Cl and Si traces from the synthesis procedure are also observed. The Al and S signals are related to the supporting carbon tape and the Al stub below; thus, they are not ascribed to our samples.

**Figure 4:**
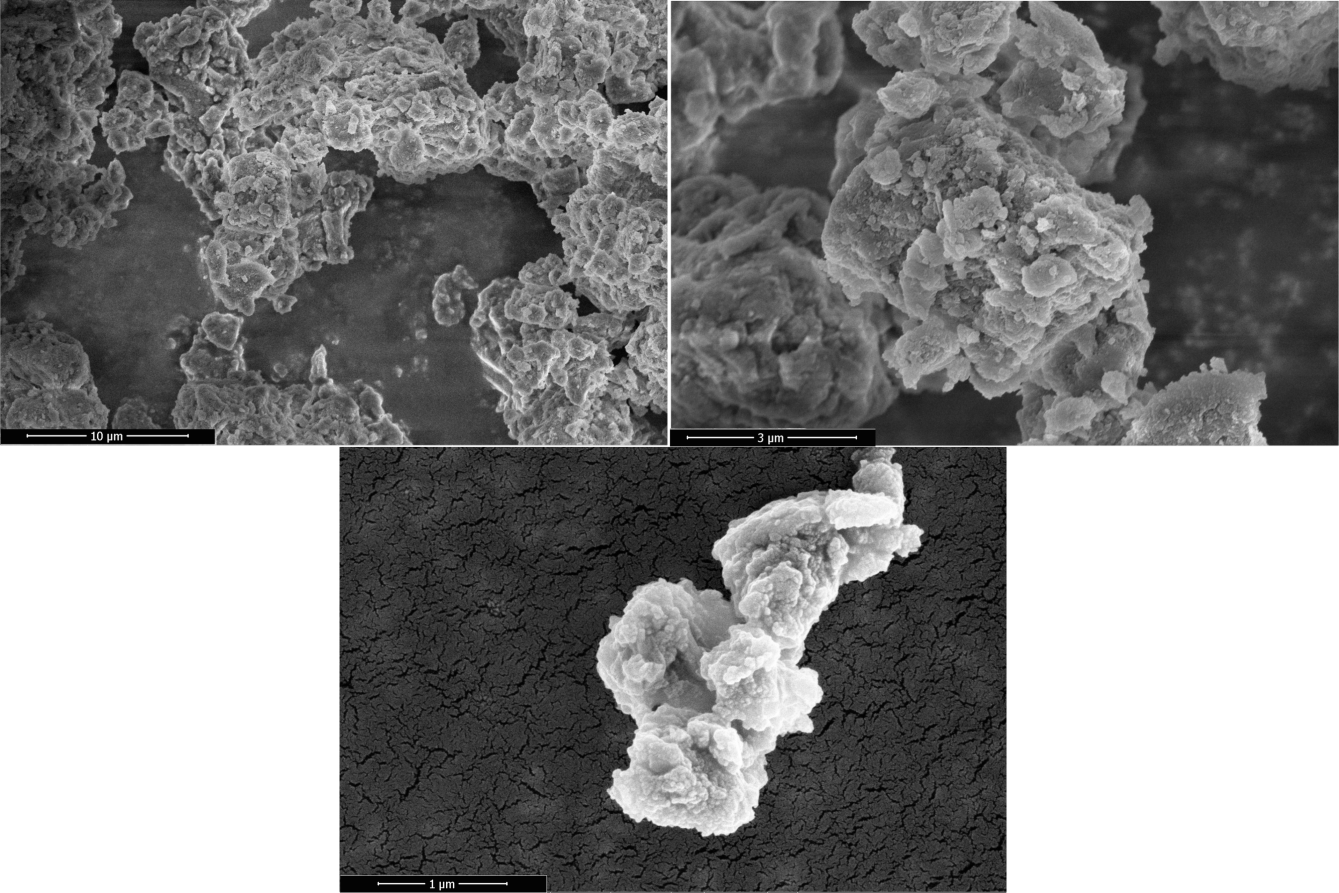
SEM analysis of AlgXCu at 10 μm, 3 μm, and 1 μm magnifications.

**Figure 5:**
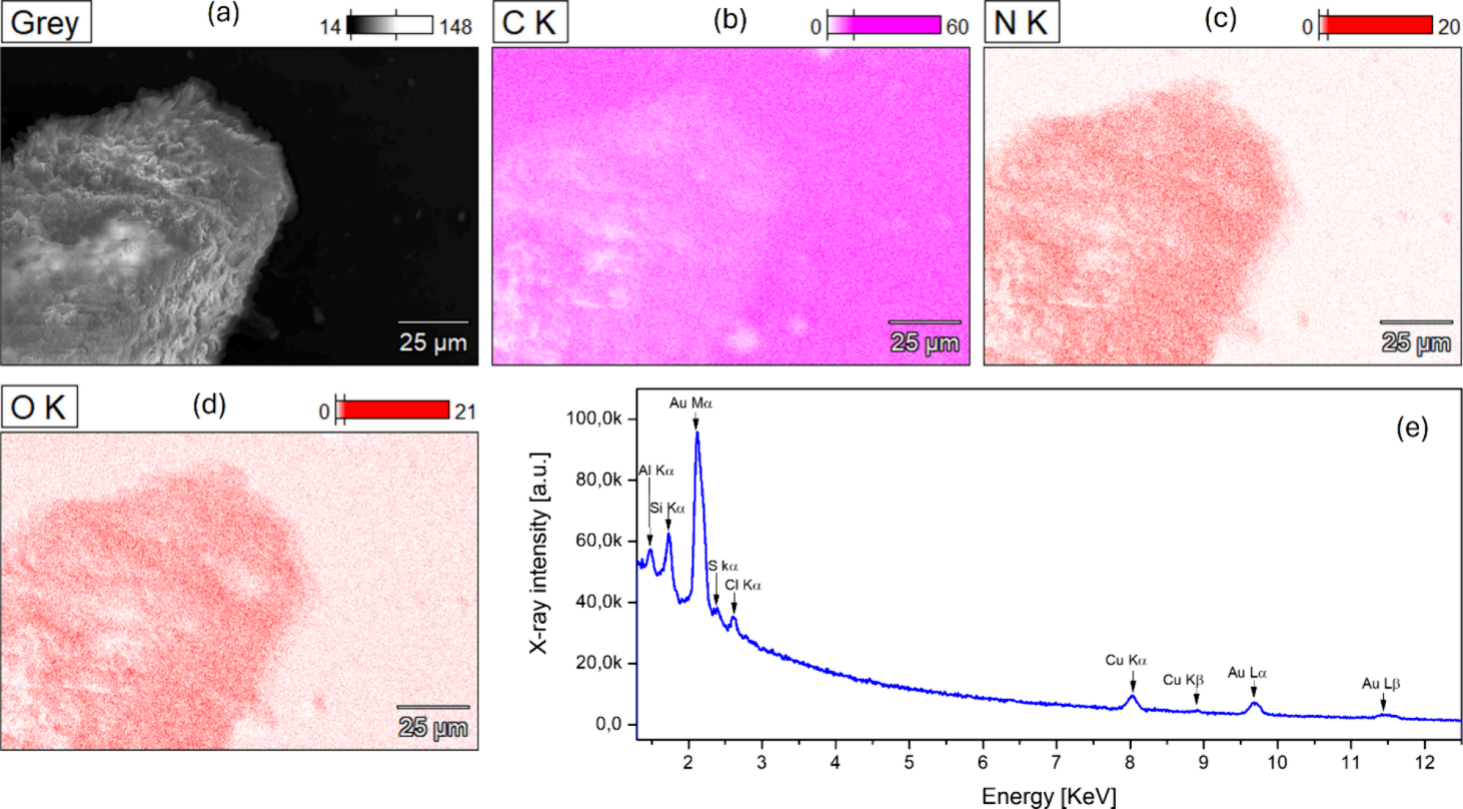
(a) EDX mapping analysis of AlgXCu: presence of (b) C, (c) N, and (d) O; (e) EDX spectrum.

### 3.3 Sensor Activity

The bare electrode (SPCE) has been extensively characterized in previous works.^19,37,40^ We therefore report the characterization of SPCE modified with AlgX, (AlgX/SPCE), and AlgXCu (AlgXCu/SPCE). AlgX/SPCE and AlgXCu/SPCE were then characterized by studying its properties using CV and EIS in 0.1 M PBS, in the presence of a standard (10 mM K_3_[Fe­(CN)_6_]). It was not possible to perform electrochemical characterization of CuCl_2_ (a precursor used in the synthesis of AlgXCu) and of Alg alone, as both the reagent and the polysaccharide does not adhere to the electrode surface and leaches into the solution; in contrast, functionalization with xanthine prevents this phenomenon, making AlgX less soluble in water and therefore more suitable for this application. The CV performed in the presence of [Fe­(CN)_6_]^3–/4–^ shows a peak-to-peak separation (ΔV) of 0.26, 0.42, and 0.14 V, thus demonstrating high-speed kinetics and electron transfer for the SPCE, AlgX/SPCE, and AlgXCu/SPCE, respectively (Figure S4a).^41^

The markedly smaller ΔEp observed for AlgXCu/SPCE indicates enhanced electron-transfer kinetics, highlighting the beneficial role of Cu incorporation in promoting faster interfacial redox processes. In contrast, the larger peak-to-peak separation observed for AlgX/SPCE indicates that the AlgX layer alone partially hinders electron transfer at the electrode interface. The incorporation of Cu within the AlgX matrix not only compensates for this inhibitory effect but reverses it, resulting in superior electron-transfer kinetics compared to both AlgX/SPCE and the bare electrode.

The cyclic voltammograms recorded in the presence of 10 mM K_3_[Fe­(CN)_6_] at different scan rates (25–500 mV s^–1^) show an increase in the anodic peak current (Ipa) with increasing scan rate. The logarithmic plot of versus exhibits a linear relationship with a slope of approximately 0.25 (Figure S4b,c).^42^

This value, being significantly lower than the theoretical slope of 0.5 expected for a purely diffusion-controlled process, indicates that the electrochemical reaction is governed by interfacial kinetic limitations. This behavior suggests that the charge-transfer process is hindered by the presence of the AlgXCu matrix, which acts as a resistive barrier, shifting the system from a diffusion-dominated regime toward one controlled by interfacial charge-transfer resistance.

The observed dependence of the anodic peak current on the scan rate suggests that mass transport of the redox probe is not significantly hindered by surface adsorption phenomena, confirming that the AlgXCu layer remains permeable to electroactive species. This behavior indicates that the incorporation of Cu into the AlgX matrix does not result in the formation of a blocking film, while preserving favorable transport properties and promoting efficient electron-transfer kinetics.

Finally, EIS analysis yielded series resistance (Rs), charge-transfer resistance (Rct), and double-layer capacitance (Cdl) values of 120 Ω, 27.9 kΩ, and 14.0 μF, respectively (Figure S4d). These results demonstrate low resistance at the electrode interface, favoring the redox reaction of the iron ion. By dividing the Cdl value by the average specific capacitance (Cs) value (40 μF/cm^2^), the electrochemically active surface area (ECSA) value was calculated to be 0.35 cm^2^.^37,43,44^

The relatively low Rct value obtained for AlgXCu/SPCE, together with the appreciable double-layer capacitance, suggests efficient charge accumulation and transfer at the electrode/electrolyte interface. Moreover, the calculated ECSA value (0.35 cm^2^) indicates an enlarged electrochemically active area compared to the geometric surface, which can be reasonably associated with the presence of Cu-based electroactive sites dispersed within the AlgX matrix.

The sensing abilities of AlgXCu/SPCE were preliminarily evaluated using CV measurements (Figure S5a,b) over increasing UA concentrations (0–1000 μM), highlighting the sensor’s strong response. As a representative example, at 10 μM UA, AlgXCu/SPCE exhibited a markedly higher oxidation current (∼2.0 μA) compared to bare SPCE (∼0.8 μA) and AlgX/SPCE (∼0.2 μA), clearly demonstrating the crucial role of Cu incorporation in enhancing the sensor response toward the target analyte (Figure S6).

Notably, the markedly lower oxidation current observed for AlgX/SPCE confirms that AlgX alone is insufficient to promote UA oxidation, further emphasizing that Cu is the key electroactive component responsible for the enhanced analytical performance of AlgXCu/SPCE.

To improve sensitivity by increasing AlgXCu/SPCE’s ability to detect low levels of UA and perform quantitative estimation, we conducted DPV measurements. Rising concentrations of UA (0 to 2000 μM) were used, and the response of AlgXCu/SPCE is reported in Figure 6a. Results, shown in Figure 6b (calibration curve), demonstrate a sensitivity of 14.28 μAμM^–1^cm^–2^ and a LOD of 0.025 μM, thus showing excellent reactivity in UA detection. Excellent repeatability at both low and high concentrations, with an RSD ≤ 1.5% was obtained.

**Figure 6:**
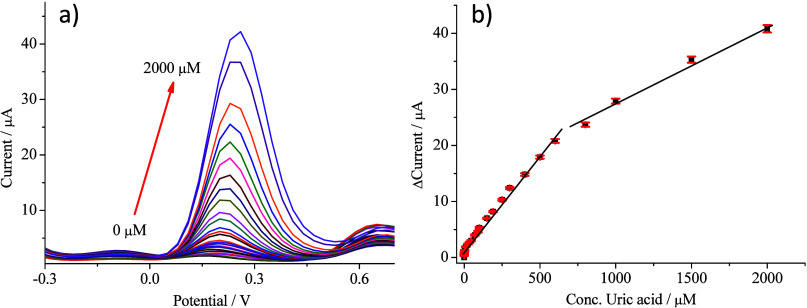
(a) DPV at different UA concentrations (0–2000 μM, initial step 0.05 μM) in 0.1 M PBS (pH 7.4); (b) calibration curve for anodic peak current (Ipa) versus the UA concentration (RSD ≤ 3.8% for five repeated whole cycles, R^2^ = 0.97368; intercept = −0.04764 ± 0.03723; slope = 5.00 ± 0.57735).

The sensor’s performance was also investigated using chronoamperometry; Figure 7a shows the current response of the AlgXCu/SPCE electrode as a function of UA concentration (using a constant applied potential of 0.35 V relative to Ag/AgCl). The corresponding calibration curve (Figure 7b) shows a linear relationship between the current and the UA concentration (μM), with a sensitivity of 3.93 × 10^–1^μA μM^–1^cm^–2^ and a LOD value of 0.015 μM.

**Figure 7:**
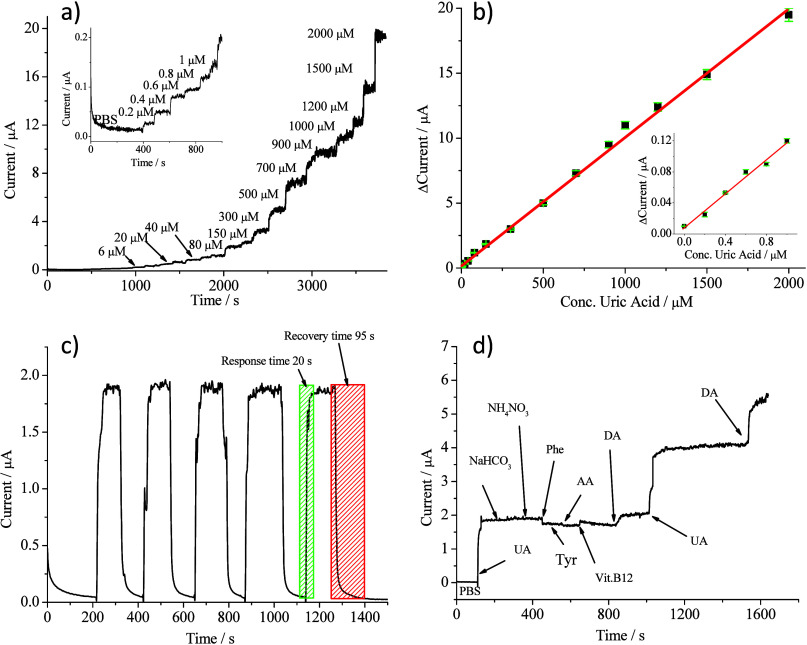
(a) Current–time response of the AlgXCu/SPCE electrode upon successive additions of UA to the 0.1 M PBS electrolyte at 0.35 V; the inset shows the response in the 0–1 μM UA. (b) Calibration line for detecting and quantifying UA; the inset shows the calibration line in the 0–1 μM UA range (R^2^ = 0.99978; intercept = −0.00233 ± 6.2361 × 10^–4^; slope = 0.1375 ± 0.00144); (c) Repeatability test toward 150 μM of UA, and response and recovery time of AlgXCu/SPCE in the absence and presence of UA; (d) Chronoamperometry response of the AlgXCu/SPCE toward UA and interferents (150 μM each).

The response time for UA concentrations in the range of 0–150 μM was 20 s, while the recovery of initial conditions performed in 0.1 M PBS (absence of UA) was 95 s (Figure 7c). These values demonstrate the excellent ability of AlgXCu/SPCE to run continuously for several cycles with high repeatability (Figure 7c; RSD of 1.1%).

Therefore, we evaluated the optimal operating pH of the new sensor and observed that the maximum potential variation (ΔV) occurred at a pH between 6 and 7.4 (Figure S7).

In addition, five freshly prepared AlgXCu/SPCE electrodes were used to measure 150 μM UA in 0.1 M PBS. All five electrodes showed identical DPV and CA responses, with an RSD of 2.2%, confirming the high reproducibility of the AlgXCu/SPCE electrode.

Crucial is to study the selectivity of this AlgXCu/SPCE sensor in the presence of interferents found in real samples, such as phenylalanine (Phe), tyrosine (Tyr), dopamine (DA), ascorbic acid (AA), vitamin B12 (Vit. B12), Na^+^, NH_4_^+^, HCO_3_^–^, and NO_3_^–^ (Figure 7d). From the chronoamperometric measurements shown in Figure 7d, AlgXCu/SPCE responds selectively to UA (150 μM), while no significant current variations are observed upon the addition of most interferents (150 μM). Dopamine (80 and 150 μM) produces a comparable current response, as expected for an electroactive species. This apparent interference is intentionally exploited in this work for the simultaneous detection of UA and DA and therefore does not represent a limitation of the proposed strategy.

Therefore, we investigated the ability of AlgXCu/SPCE to differentiate and detect both DA and UA. Initial simultaneous detection experiments performed at identical UA and DA concentrations were carried out under standardized model conditions to evaluate peak separation and the intrinsic selectivity of the sensor. These conditions were specifically selected for analytical characterization and do not reflect the physiological concentration levels found in actual biological fluids, in order to demonstrate that the sensor is capable of detecting both analytes, as a proof of concept.

Figure 8a shows two well-separated current peaks, allowing independent electrochemical resolution of the two analytes and a simultaneous linear response (Figure 8b) over the 0–1000 μM concentration range for both DA and UA.

**Figure 8:**
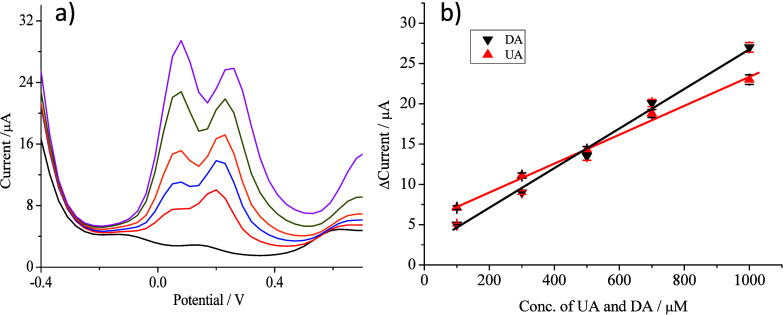
(a) DPV analyses using the AlgXCu/SPCE sensor for the determination of DA and UA (0−1000 μM for each, (nonphysiological conditions) in PBS (0.1 M); (b) calibration curve for anodic peak current (*I*_pa_) versus the UA (red arrowhead) and DA (black arrowhead) concentration.

The temporal stability of the AlgXCu/SPCE sensor was evaluated by testing the same device at 0, 7, 15, and 30 days after preparation. The sensor maintained consistent performance throughout the testing period, with minimal signal variation (RSD ≤ 2.2%; Figure S8).

The electrochemical oxidation of DA and UA is facilitated by a synergistic mechanism involving both the AlgX matrix and the coordinated centers. While the AlgX scaffold acts as a macromolecular ligand that preconcentrates the analytes and prevents electrode fouling through its porous network, the immobilized ions serve as the primary electrocatalytic sites. Based on literature for similar copper-modified systems,^20,45–47^ we hypothesize that the mechanism involves the redox couple acting as an interfacial mediator. This mediation occurs through the formation of transient coordination complexes between copper and the functional groups of the analytes, specifically the catechol moieties of DA and the nitrogen/carbonyl sites of UA. This possible "molecular bridge" facilitates a faster interfacial electron transfer to the electrode surface, significantly lowering the overpotential and allowing for the clear simultaneous resolution of both oxidation peaks.

To investigate the detection behavior of AlgXCu/SPCE under conditions mimicking real samples, synthetic saliva was used.

Measurements were performed before and after adding 0.1 mL of synthetic saliva to 3.9 mL of PBS aqueous solution (1:40). Figure 9a shows a current variation of 0.5 μA after adding synthetic saliva. From the calibration line (Figure 6b), UA concentrations of 1 μM were tested, and using a 1:40 dilution, we found a concentration of 4.0 × 10^–5^ mol/L in synthetic saliva (40 μM), confirming the amount of UA present in the sample.

**Figure 9:**
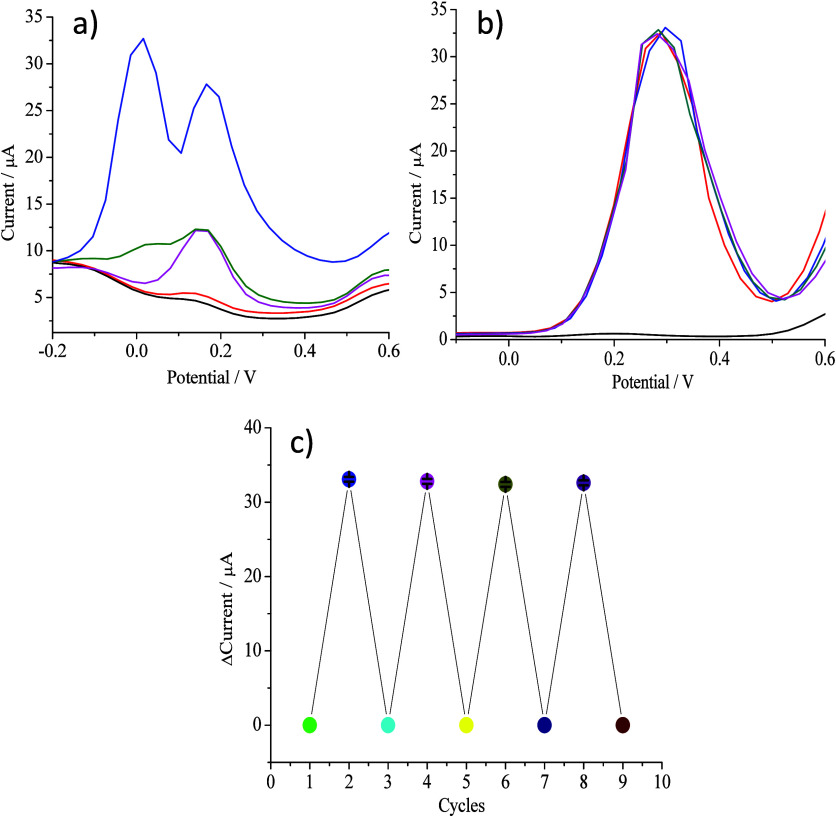
(a) DPV analyses to determine 100/0 (magenta curve), 100/100 (dark yellow curve), and 1000/1000 (blue curve) μM of DA/UA, added to PBS 0.1 M (3.9 mL, black curve) and the synthetic saliva (0.1 mL, red curve). (b) DPV of AlgXCu/SPCE for synthetic urine, (c) DPV measurements using the same AlgXCu/SPCE sensor by cycling five times in the absence and presence of a synthetic urine sample (UA = 1400 μM).

Subsequently, recovery experiments were performed by spiking the diluted synthetic saliva samples with 40 and 400 μL of a 10 mM DA and UA standard solution (corresponding to 4 × 10^–7^ and 4 × 10^–6^ mol, i.e., nominal concentrations of 100 and 1000 μM in the electrochemical cell). These experiments were designed to assess sensor response and recovery under controlled analytical conditions rather than to mimic physiological DA levels (Figure 9a and Table 3).^41,48^

**Table 3:** AlgXCu/SPCE Sensor Response to UA and DA in Synthetic Matrices

**Sample**	**Added** UA/DA (μM)	**Measured** UA/DA (μM)	**Recovery (%)**	**RSD (%)**
PBS 0.1M	100/100	100/100	100/100	1.9/2.1
	1000/1000	1000/1000	100/100	1.8/1.9
Saliva	100/0	104/0	104.0/100	2.2
	100/100	104.2/105.0	104.2/105.0	2.4/2.0
	1000/1000	1013.0/1010.0	101.3/101.0	1.8/2.0
Urine	1400/0	1370.0/0	97.9/100	1.0

In addition, we measured UA concentrations in synthetic urine samples containing a known UA concentration (1.4 mM), and Figure 9b shows excellent repeatability with an RSD ≤ 1%. The recovery data reported in Table 3 show recoveries of 97.9–105.0% for UA and DA in saliva and urine, confirming the good sensing capabilities of AlgXCu/SPCE.

Finally, Figure 9c shows the ability of AlgXCu/SPCE to undergo continuous monitoring cycles of UA in urine, with exceptional stability and repeatability (RSD = 1.0%).

To better approximate physiological conditions and account for the strong concentration asymmetry between UA and DA in saliva, DA was subsequently analyzed in the nanomolar range (0.1–0.3 μM), while UA was maintained at higher concentrations (25 and 150 μM), consistent with reported salivary values (Figure 10). It is important to emphasize that the successful resolution of 0.1 μM DA in the presence of 150 μM UA provides significant evidence of the sensor’s resistance to concentration asymmetry and paves the way for future clinical applications where high peak separation is a fundamental prerequisite for reliable detection, thereby confirming the proof of concept of the sensor’s applicability.

**Figure 10:**
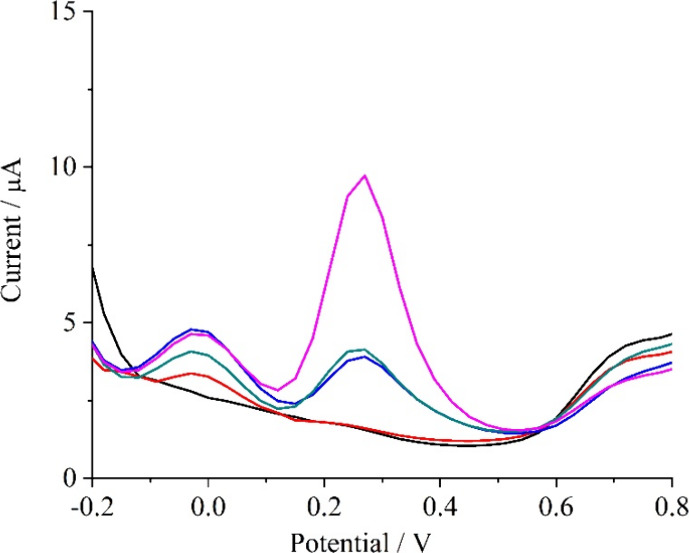
DPV analyses using the AlgXCu/SPCE sensor for the simultaneous determination of DA 0 (black line), 0.1 (red line), 0.2 (green line), and 0.3 (blue, and pink lines) μM and UA 0 (black line), 25 (green, and blue lines), and 150 (pink line) μM in synthetic saliva samples.

The good ability of our sensor to detect uric acid is compared with that of numerous copper-based sensors in Table 4, which also compares the percentage by weight of copper contained in the various sensors. The comparative table clearly shows that, although AlgXCu contains 4.8% copper, it has a low limit of detection (LOD). In addition, our sensor is simple and easy to manufacture, key characteristics for its potential industrial-scale production. Finally, its low copper content and the use of natural substrates allow us to classify it as an eco-friendly sensor, an important feature among the sensors listed (Table 4).

**Table 4:** Comparison of the Detection Performance and Cu Content (% wt) of AlgXCu with Those of the Copper-Based Systems Previously Described for the Detection of Uric Acid

**Electrode materials**	**Cu content** (% wt)	**LOD μM**	**Reference**
AlgXCu	∼4.8	0.025	This work
GC/CuO/Chitosan	∼53.0	0.028	^49^
NT Cu-MOF/CPE	∼25.0	0.1	^50^
Cu-BTC	∼3.1	0.2	^51^
Cu HAp FTO	∼6.3	0.5	^52^
CuO nanorice/GCE	∼79.0	1.2	^53^
CuO/GCE	∼80.0	0.6	^54^
UOx/CuO/CPE	∼24.0	8.82	^55^
([Cu(PhCOO)(H_2_O)_2_]·PhCOO·H_2_O)n/GPE	∼0.8	4.6	^56^
CNT/CCNT@Cu-ZIF-67	∼5.0	0.32	^57^
CuBi_2_O_4_	∼11.6	1.23	^58^
Au–Cu_2_O/rGO	∼40.0	6.5	^59^
hnp-PtCu	∼10.0	5.7	^60^
Au@Cu-MOF	∼25.0	10.36	^61^
CS-GSH-CuO/GCE	∼15.0	0.27	^45^
ZnO–CuxO/polypyrrole	∼15.0	0.2	^62^
Heart/dumbbell-like CuO	∼80.0	0.5	^63^
CuNPs/Cu(II)-PDA/Gr	∼15.0	6.2	^46^
ZnO NRs-CuO NSs/GCE	∼5.0	0.28	^64^
CuNW	100	2.0	^65^
Cu(II)-activated mp20@ZIF-8/rGO/SPCE	∼0.2	0.21	^66^

## 4 Conclusions

The developed AlgXCu/SPCE platform represents a significant breakthrough in electrochemical sensing, successfully merging high analytical performance with an unprecedented eco-compatibility profile, attributed mainly to its design and fabrication. The sensor is built on a natural, eco-biocompatible polymer (alginate), with its functionalization (AlgX synthesis) achieved via a truly sustainable synthetic process that uses water as the primary solvent and avoids harsh chemicals, ensuring low environmental impact from the outset. Crucially, the final active material, AlgXCu, exhibits a low metal catalyst concentration (4.8% Cu^2+^); this factor, combined with the natural origin of the substrate, allows us to definitively classify it as an environmentally friendly sensor, a key differentiator from many high-performance electrochemical devices. Despite this minimal copper usage, the AlgXCu sensor achieves an outstanding LOD (0.025 μM), making it highly competitive and even surpassing many copper-based sensors reported in the literature, while also demonstrating good stability, high recovery in complex biological matrices (saliva and urine), and reliable simultaneous detection of UA and DA. In conclusion, the analytical performance demonstrated in synthetic matrices identifies this sensor as a promising proof-of-concept for the simultaneous detection of uric acid and dopamine. While these results suggest a potential interest for Lesch–Nyhan syndrome monitoring, we recognize that the present study is limited to controlled environments. Extensive clinical validation using real human samples is mandatory before any diagnostic applicability can be claimed. Nevertheless, the simplicity, cost-effectiveness, and green synthesis of the AlgXCu platform provide a solid foundation for future analytical developments and potential industrial scalability.

## Supplementary Material



## Notes

(Figure S1) Picture of AlgX; (Figure S2) ^1^H NMR of Alg; (Figure S3) ^1^H NMR of AlgX; (Table S1) operating condition for NexION 5000 ICP-MS; (Figure S4) (a) CV AlgXCu/SPCE in the presence of 10 mM K_3_[Fe­(CN)_6_] and 0.1 M PBS at a 50 mV/s scan rate, in the −0.4 to 1.1 V potential window, CV of AlgXCu/SPCE in the presence of 10 mM K_3_[Fe­(CN)_6_] at different scan rates from 25 to 500 mV/s in 0.1 M PBS (pH 7.4), (c) plot of ln­(*I*_pa_) vs ln­(υ) (R^2^ = 0.99369; intercept = 1.44378 ± 0.0165; slope = 0.24805 ± 0.00747), (d) Nyquist plots of AlgXCu/SPCE and the equivalent circuit in the inset; (Figure S5) (a) CV at different UA concentrations (0–1000 μM, initial step 1 μM) in 0.1 M PBS (pH 7.4), (b) calibration curve for anodic peak current (*I*_pa_) versus the UA concentration (SD ≤ 1.5 for five repeated whole cycles, R^2^ = 0.97727; intercept = 1.41885 ± 0.0414; slope = 0.05959 ± 0.00639); (Figure S6) electrochemical response with 10 μM UA in 0.01 M PBS; (Figure S7) DPV for AlgXCu/SPCE upon 100 μM uric acid at different pH values (4.5, 5.0, 6.0, 7.4, 8.0, and 10.0; RSD ≤ 2.9%); and (Figure S8) DPV measurements using the same AlgXCu/SPCE sensor after simultaneous addition of 100 μM of DA and UA at different times (PDF)
