# Supplementary material for: Sustainable Xanthine-Grafted Alginate Biosensing Platform for Metabolic Disorder Diagnostics
Source: ACS Omega. 2026 Jun 1;11(23):34467–79. doi: 10.1021/acsomega.6c02447 (PMC13280848; doi:10.1021/acsomega.6c02447)
Supplement: Supplementary file 1 [file ao6c02447_si_001.pdf]

Supporting Information

Sustainable Xanthine-Grafted Alginate Biosensing Platform for Metabolic Disorder Diagnostics

Angelo Ferlazzo [a], Erika Saccullo [b,c], Giulia Sambataro [b], Elena Bruno [d], Manuel David Montaño [e], Vincenzo Abbate [f], Salvatore Failla [a], Venerando Pistarà [b], Antonino Gulino [a], Antonio Rescifina [b], Vincenzo Patamia [b,*], Giuseppe Floresta [b,*]

[a] *Department of Chemical Sciences, University of Catania, Viale Andrea Doria 6, 95125 Catania, Italy*

[b] *Department of Drug and Health Sciences, University of Catania, Viale Andrea Doria 6, 95125 Catania, Italy*

[c] *Department of Biomedical and Biotechnological Sciences (Biometec), University of Catania, Via Santa Sofia 97, 95123 Catania, Italy.*

[d] *Department of Physics and Astronomy "Ettore Majorana", University of Catania, via S. Sofia 64, 95123 Catania, Italy, and CNR-IMM, Via Santa Sofia 64, Catania, 95123, Italy.*

[e] *London Metallomics Facility, King's College London, London, UK.*

[f] *Department of Analytical, Environmental & Forensic Sciences, Faculty of Life Sciences & Medicine, King's College London, London, UK*

Corresponding authors

e-mail: vincenzo.patamia@unict.it, giuseppe.floresta@unict.it

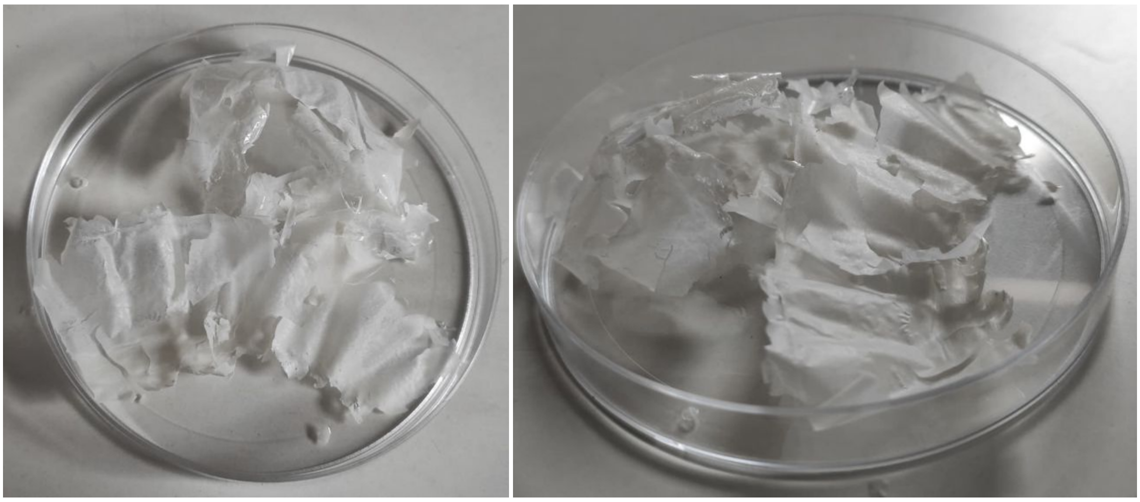

Figure S1. AlgX.

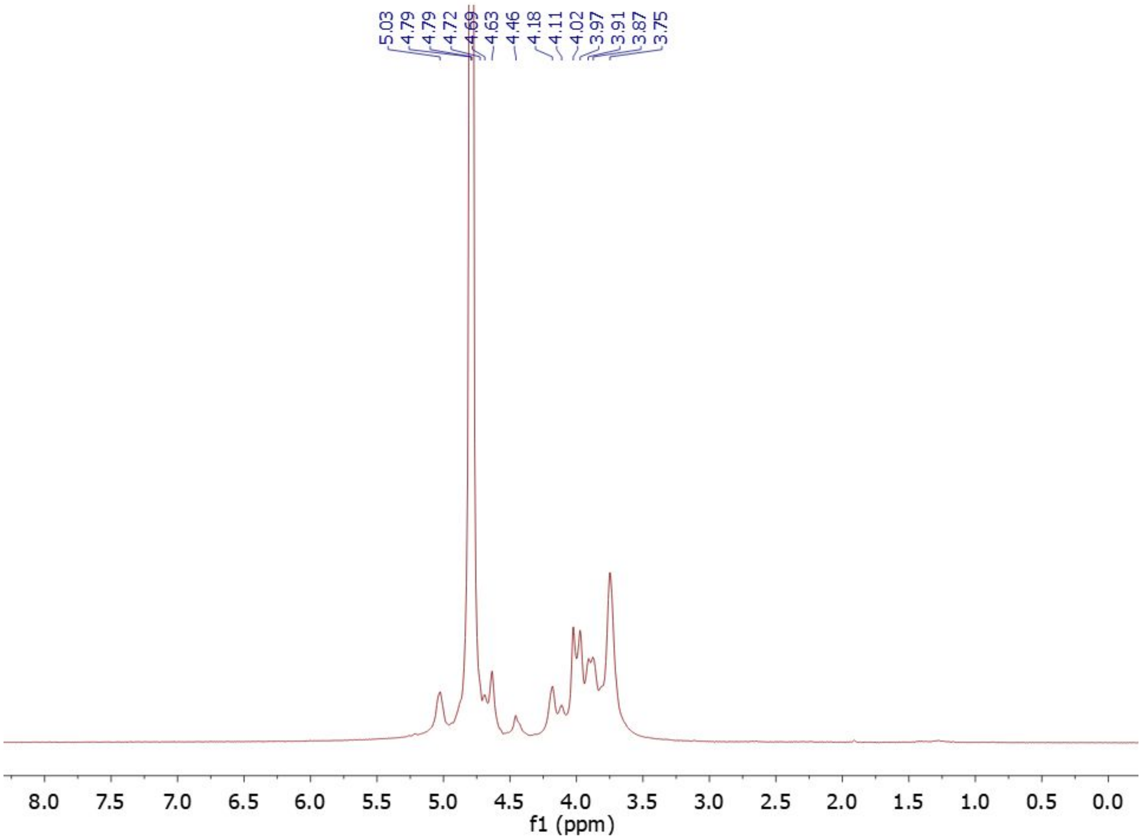

Figure S2. ^{1}H NMR of Alg.

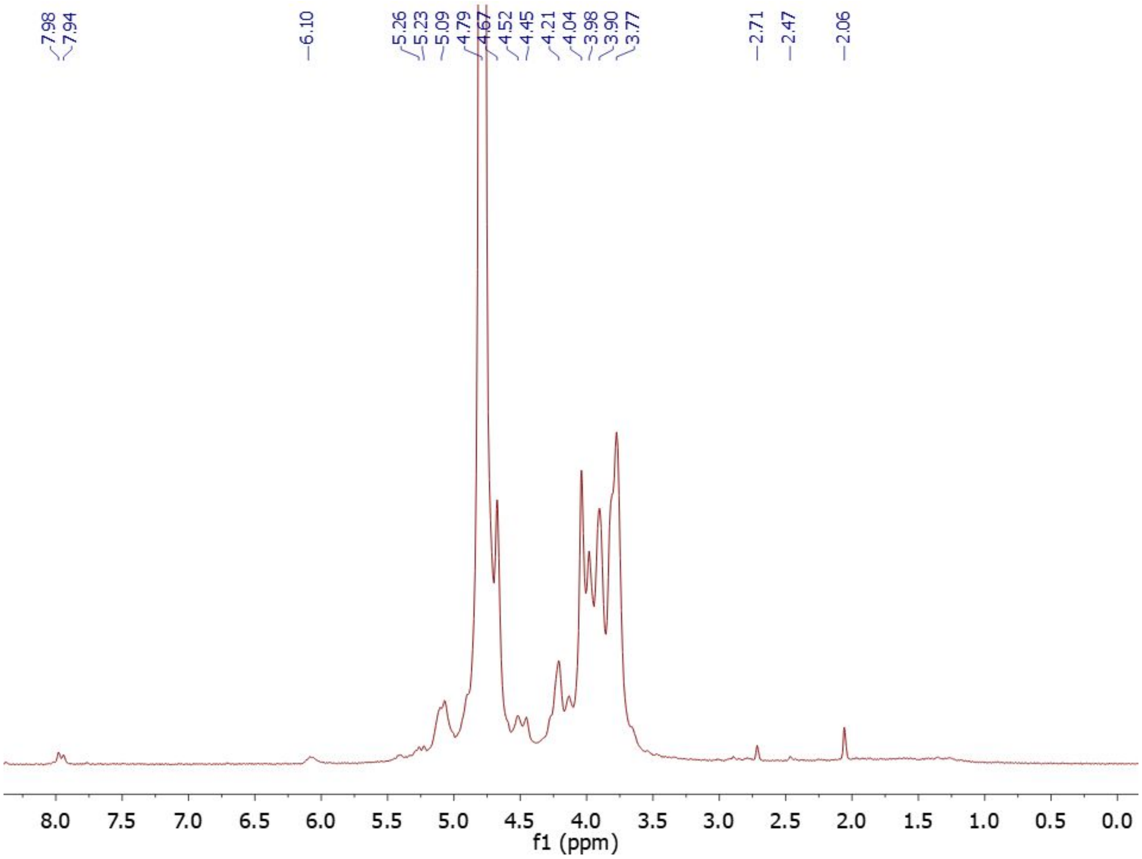

Figure S3. ^{1}H NMR of AlgX.

Table S1. Operating condition for NexION 5000 ICP-MS.

Instrument Parameter	Value
**ICP RF Power**	1600 W
**Neublizer Gas Flow**	0.94 L/min
**Auxiliary Gas Flow**	1.3 L/min
**Plasma Gas Flow**	18 L/min
**Ammonia DRC Gas Flow**	0.6 mL/min
**QID Fixed Voltage**	–13 V
**Sample Flow Rates**	0.28 mL/min
**Dwell Time**	100 ms
**Analog Stage Voltage**	–1875 V
**Pulse Stage Voltage**	1650 V

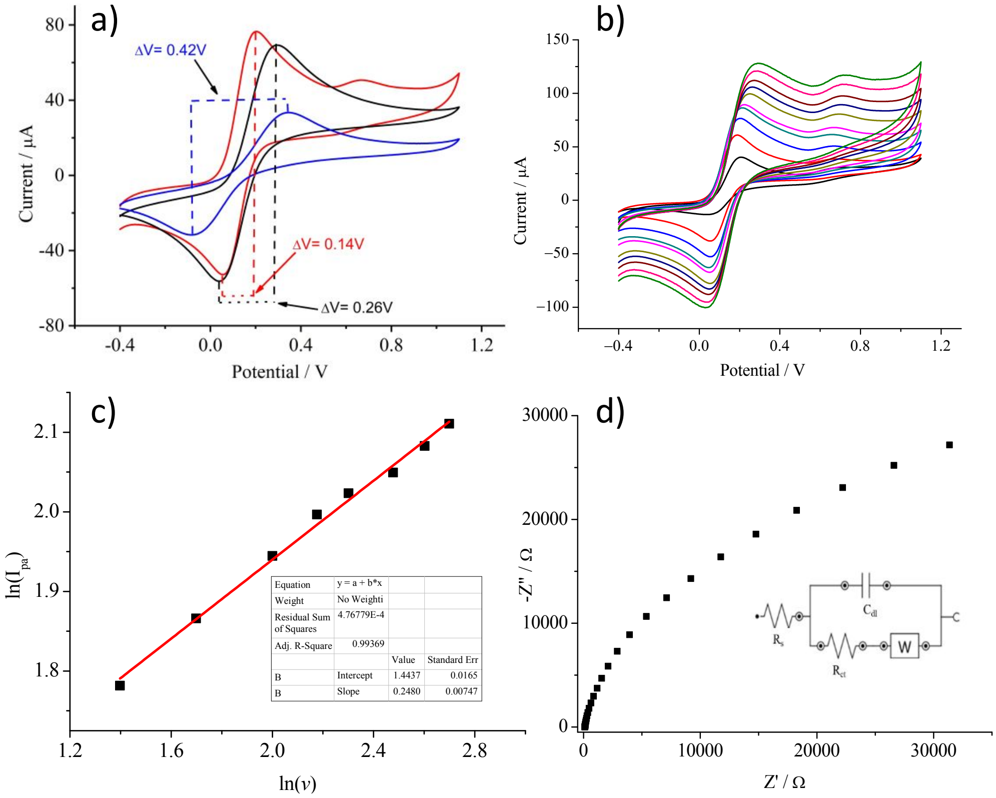

Figure S4. (a) CVs SPCE (black line), AlgX/SPCE (blu line), and AlgXCu/SPCE (red line) in the presence of 10 mM $K_3[Fe(CN)_6]$ and 0.1 M PBS at a 50 mV/s scan rate, in the −0.4 to 1.1 V potential window. (b) CV of AlgXCu/SPCE in the presence of 10 mM $K_3[Fe(CN)_6]$ at different scan rates from 25 to 500 mV/s in 0.1 M PBS (pH 7.4). (c) Plot of ln(I_{pa}) vs ln(υ) (R^2 = 0.99369; intercept = 1.44378 ±0.0165; slope = 0.24805 ±0.00747). (d) Nyquist plots of AlgXCu/SPCE and the equivalent circuit in the inset.

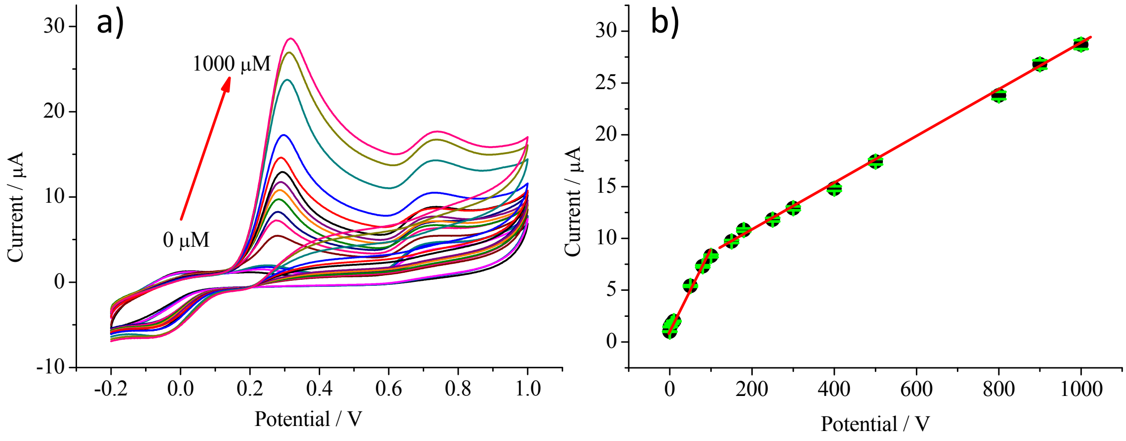

Figure S5. (a) CV at different UA concentrations (0–1000 μM, initial step 1 μM) in 0.1 M PBS (pH 7.4); (b) calibration curve for anodic peak current (I_{pa}) *versus* the UA concentration (SD ≤1.5 for 5 repeated whole cycles, R^2 = 0.97727; intercept = 1.41885 ±0.0414; slope = 0.05959 ±0.00639).

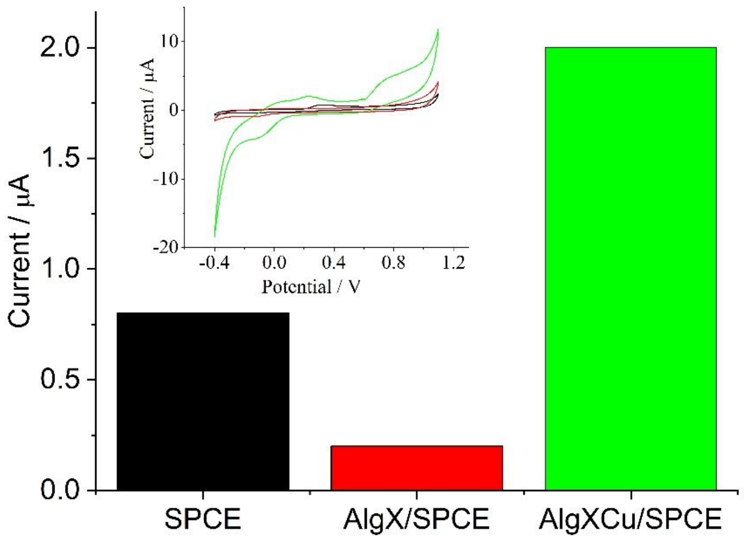

Figure S6. Electrochemical response with 10 μM UA in 0.01 M PBS at a scan rate of 50 mV/s of SPCE (black line), AlgX/SPCE (red line), and AlgXCu/SPCE (green line) in the -0.4–1.1 V potential window, electrochemical behaviors in the insert.

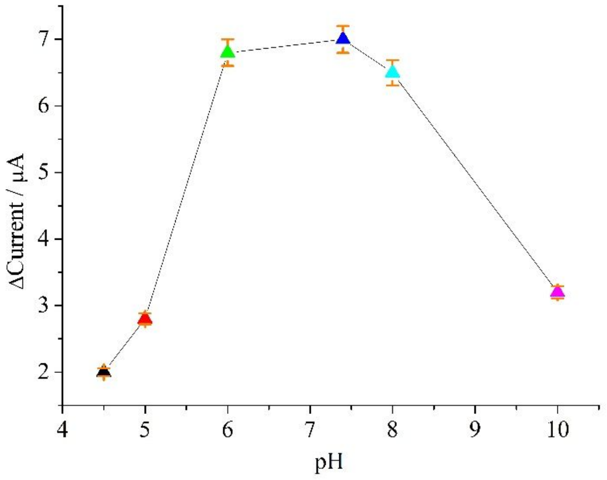

Figure S7. DPV for AlgXCu/SPCE upon 100 μM uric acid at different pH values (4.5, 5.0, 6.0, 7.4, 8.0 and 10.0, RSD ≤2.9%).

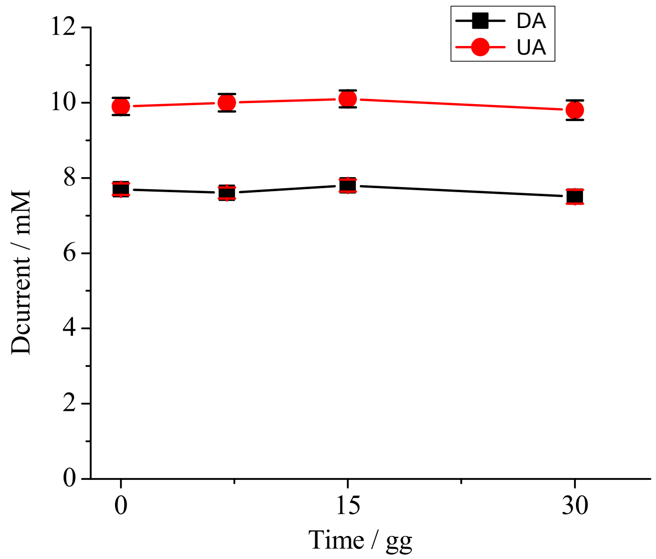

Figure S8. DPV measurements using the same AlgXCu/SPCE sensor after simultaneous addition of 100 µM of DA and UA different times.
